# Bioelectrical impedance vector analysis in the critically ill: cool tool or just another ‘toy’?

**DOI:** 10.1186/s13054-015-1110-7

**Published:** 2015-11-11

**Authors:** Lui G. Forni, Julia Hasslacher, Michael Joannidis

**Affiliations:** Department of Intensive Care Medicine, Royal Surrey County Hospital NHS Foundation Trust, Egerton Road, Guildford, Surrey GU2 7XX UK; Surrey Perioperative Anaesthesia Critical care collaborative Research group (SPACeR) and Faculty of Health Care Sciences, Duke of Kent Building, University of Surrey, Guildford, Surrey GU2 7TE UK; Division of Intensive Care and Emergency Medicine, Department of Internal Medicine, Medical University Innsbruck, Anichstraße 35, A-6020 Innsbruck, Austria

## Abstract

Assessment of volume and hydration status is far from easy and therefore technology such as bioelectrical impedance vector analysis (BIVA) may complement our examination techniques. This study highlights the fact that clinical assessment of volume balance and BIVA may correlate, but whether the routine use of BIVA will avoid significant volume overload in the critically ill remains unknown. Further studies are needed but at the moment appear a little way off.

In the current issue of *Critical Care*, Jones et al. [1] investigated the use of bioelectrical impedance vector analysis (BIVA) for estimation of volume status in critically ill patients. Although one of the basic tenets of clinical examination, assessment of volume status can prove to be somewhat challenging. Volume assessment is confounded by parameters such as venous capacitance, which is difficult to quantify accurately, and hydration status, which is even more complex. Simplistically, volume status reflects the balance between extracellular and intracellular volume. Both volume depletion and overload are associated with increased mortality in the critically ill. Volume overload may be defined on clinical examination (although this may not be appreciated until several litres of volume expansion have occurred) or as fractional increase of body weight. Accurate weight is often difficult to obtain in critically ill patients, however, and different signals are obtained based on the definition of weight changes, even with respect to outcome [2]. Hydration status—that is, quantifying the true water status of an individual—may be described as a positive water balance (hyperhydration: water excess) or a negative water balance (hypohydration: water deficit) [3]. With dehydration being the loss of water from the body and rehydration being the process of gaining body water (note: water not volume!) (Fig. 1), it follows that euhydration is a state of being ‘in water balance’ which, of course, is not a steady state. Euhydration is rather a dynamic condition that may be even found in a situation in which electrolytes/osmolytes are lost from the body but water is retained, resulting in a relatively hypovolemic but euhydrated patient [4]. The gold standard for estimating the body water content is the use of tracers such as deuterium oxide (D_2_O) [5]. Tracers are distributed within the order of 3–4 hours following oral loading, correction can be made for exchange with non-aqueous hydrogen and total body water can be measured with a precision and accuracy of 1–2 %. Clearly this is impractical in the ICU.

**Fig. 1:**
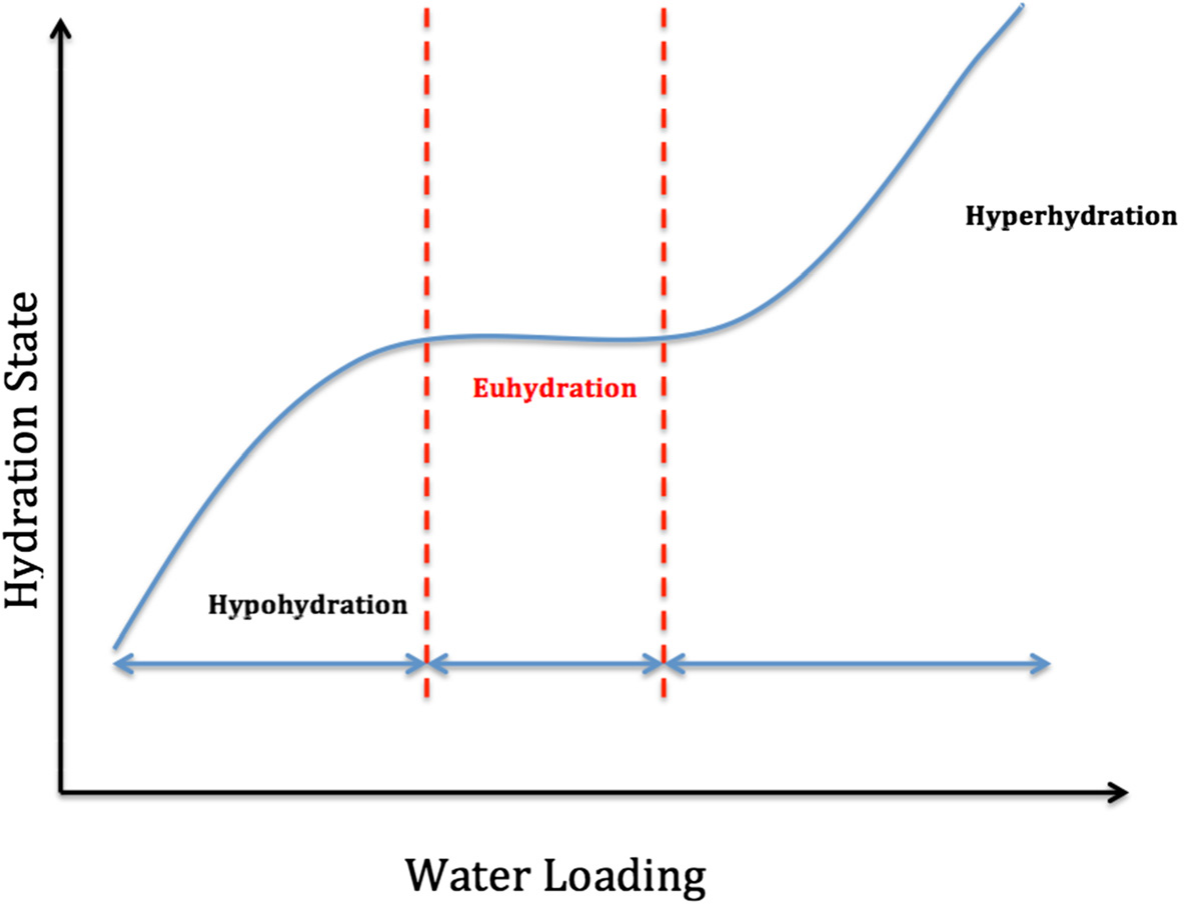
Relationship between water loading and hydration status. Idealised relationship between the observed hydration (presumed volume) status and water (fluid) loading

Bioelectrical impedance analysis was originally introduced as a tool for assessing body composition and nutritional status, but early studies highlighted some limitations of this technique with variation in electrolyte levels, acute changes in hydration status and problems with some of the standard equations employed creating some disaffection [6, 7]. BIVA systems measure hydration status, or total body water as a percentage fat-free body mass. BIVA measures whole-body impedance as a combination of resistance (R) and reactance (Xc). From this combination, the arc tangent of Xc/R is calculated (the phase angle) which represents the phase difference between voltage and current. Data are then plotted on a nomogram derived from healthy subjects. BIVA to estimate volume status has been successfully used in dialysis patients [8], where it appears to be an independent predictor of survival [9], and patients with acute heart failure [10, 11]. Studies are also available for critically ill patients [12–14].

In the present study by Jones et al. [1], 344 measurements in 61 mechanically ventilated patients were performed with 23 % determined as dehydrated, 36 % normally hydrated and 41 % overhydrated on admission to the ICU. This is the first study in which clinicians were blinded to the results of the BIVA measurements and, reassuringly, clinical assessment resulted in an increase in the subsequent cumulative fluid balance in patients who were deemed dehydrated and a decrease in overhydrated patients. Although a statistically significant correlation between the changes in fluid balance and the changes in BIVA-defined hydration could be established, sensitivity of this method in this specific cohort must be described as somewhat disappointing at best. Changes in volume status between 1 and 2 l could not be detected at all, and even changes of more than 2 l in cumulative fluid balance were reflected by BIVA only in overhydrated patients where fluid removal was achieved. However, the physicians, relying on clinical evaluation, did seem to react in an appropriate manner in terms of fluid prescription without knowing the BIVA values, in that they aimed for, and achieved, a negative fluid balance. Interestingly, lactate levels correlated with volume status and changes in fluid balance, but not with BIVA assessment. This leads us to possible limitations for BIVA in the critically ill. First of all, estimation of hydration status is related to fat-free mass, which basically means muscle mass (in the limbs). Whereas the limbs contribute roughly 90 % to whole body impedance, only 6–12 % are contributed by the trunk which, however, provides roughly 50 % of the body weight and stores most of the surplus volume [15]. It is well recognised that in critical illness proteolysis results in rapid loss of muscle mass within the first days. Secondly, fluid shifts in the critically ill may be considerable and not necessarily isotonic. Third, it has been demonstrated that brain natriuretic peptide as a biomarker of volume overload does not well correlate with BIVA in critically ill patients treated with continuous renal replacement therapy (CRRT) [13], indicating that hydration status alone may not be sufficient to guide fluid therapy or to predict outcome.

In summary, can BIVA guide fluid management in critically ill patients? As pointed out by Jones et al., this can only be addressed in well-designed interventional studies particularly with regard to the patient population. Given these preliminary results it seems unlikely that BIVA will play a major role in the critically ill level 3 patient with sepsis where rapid fluid shifts are occurring or in the unstable postoperative patient, but the technique may inform in less acute situations such as renal replacement therapy in a step-down unit.

## Abbreviations

BIVA: Bioelectrical impedance vector analysis
CRRT: Continuous renal replacement therapy
R: Resistance
Xc: Reactance
